# Colour Evaluation, Bioactive Compound Content, Phenolic Acid Profiles and in Vitro Biological Activity of Passerina del Frusinate White Wines: Influence of Pre-Fermentative Skin Contact Times

**DOI:** 10.3390/molecules21070960

**Published:** 2016-07-22

**Authors:** Katya Carbone, Luciano Fiordiponti

**Affiliations:** Consiglio per la ricerca in Agricoltura e l’Analisi dell’Economia Agraria—Fruit Tree Research Centre; Via di Fioranello, 52-00134 Rome, Italy; lucianofiordiponti@gmail.com

**Keywords:** antioxidants, phenolic acids, maceration, antiradical capacity

## Abstract

Passerina del Frusinate is an autochthonous wine grape variety, which grows in the Lazio region that is currently being evaluated by local wine producers. In this study, colour properties (CIELab coordinates), bioactive compounds (total polyphenols and flavan-3-ols), HPLC-DAD phenolic acid profiles and in vitro biological activity of monovarietal Passerina del Frusinate white wines and the effect of different maceration times (0, 18 and 24 h) were evaluated based on these parameters. Results highlighted statistically significant differences for almost all analysed parameters due to a strong influence of the pre-fermentative skin contact time. The flavan content of macerated wines was six times higher than that of the control, while total polyphenols were 1.5 times higher. According to their phytochemical content, macerated wines showed the highest antiradical capacity tested by means of DPPH^•^ and ABTS^+•^ assays. Besides, prolonged maceration resulted in a reduction of CIELab coordinates as well as of the content of phenolic substances and antiradical capacity. Among the phenolic acids analysed, the most abundant were vanillic acid and caffeic acid; the latter proved to be the most susceptible to degradation as a result of prolonged maceration. Passerina del Frusinate appears as a phenol-rich white wine with a strong antioxidant potential similar to that of red wines.

## 1. Introduction

Passerina del Frusinate (PF) is a recognised geographical indication (RGI) for wine originating in the southwest of Rome, in the region of Lazio. Passerina is an old white grape variety of *Vitis vinifera* L., which grows in central Italy. It is currently widespread in the Marches, particularly in Piceno, Abruzzo, Emilia Romagna and Lazio, almost exclusively in the province of Frosinone, where it is known as Passerina del Frusinate cv. Although PF has been replaced for a long period in cultivation with other varieties, such as Trebbiano, which is more vigorous and productive but of lower quality, it is now at the centre of the renewed interest of local vine growers, thanks to the excellent quality of its RGI wines. In the province of Frosinone, the vinification of PF grapes occurs both with and without skin contact. Pre-fermentative skin contact is an increasingly widespread alternative technique for the production of white wines adopted to extract the phenolics responsible for the aged character of wine [1].

It is well known that phenolic compounds contribute to the sensory characteristics of wine, such as colour, flavour and astringency [2]. Moreover, several studies have highlighted the health properties of polyphenols found in grapes and wine, which are responsible for most of their antioxidant properties [3]. Wine (especially red wine) contains a plethora of phenolics such as phenolic acids, flavonoids, tannins, stilbenes and coumarins, and for this reason, at a moderate level of consumption, may be considered a good functional food [4]. In this regard, several epidemiological studies have demonstrated a direct association between the reduced risk of cardiovascular diseases and moderate alchohol consumption [4]. Sacanella et al. (2007) reported a reduction of some inflammatory biomarkers associated with coronary heart disease after wine consumption, which reached 51% in the case of white wine’s somministration [5]. Phenolic compounds are marked by a broad spectrum of health-promoting functions such as antioxidants, blood pressure- or blood sugar-influencing substances, or agents with anticarcinogenic, immunity-supporting, antibacterial, antifungal, antiviral, cholesterol-lowering, antithrombotic or anti-inflammatory effects [4,6]. In white winemaking, maceration is generally used to enrich wine in aroma compounds. However, wine practices also affect the final content of polyphenols in wine [4], and this aspect is very important to consider as polyphenols not only enhance the functional properties of wine but also strongly affect its sensorial traits. In this regard, it is fundamental to keep the phenolic content in a range that does not compromise the wine’s acceptability by consumers [7] and, consequently, determine the correct length of maceration during vinification. Among polyphenols, the most important compounds in white wines, both in terms of quantity and ability to participate in redox reactions, are non-flavonoid phenols: mainly hydroxybenzoic (HBA) and hydroxycinnamic (HCA) acids [8,9]. In particular, it has been reported that cinnamic acids are precursors of volatile aroma compounds and are also associated, with flavan-3-ols, with the wine browning process [10]. They also exert important antioxidant properties and have potential osteogenic effects, particularly *p*-coumaric acid [11]. To date, no information is available on the quality traits (e.g., colour properties and phytochemical content) and antioxidant potential of Passerina del Frusinate wine. In addition, since the vinification of PF grapes can occur with or without skin maceration affecting the bioactive molecule composition and the final colour of the produced wines, the present study aimed at investigating the bioactive compound content, antioxidant potential and phenolic acid profile of autochthonous Passerina del Frusinate white wines as well as their potential influence on the colour of wines as a function of the pre-fermentative skin contact times.

## 2. Results and Discussion

### 2.1. Color Evaluation

**Figure 1:**
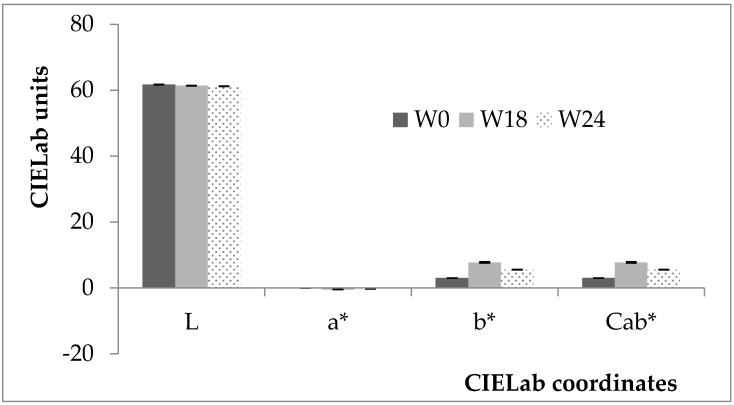
Influence of pre-fermentative skin contact times on the wine colour properties. W0: non-macerated wines; W18: maceration length 18 h; W24: maceration length 24 h. Data are expressed as CIELab coordinates (mean ± SE). Experimental number: 24 samples.

Figure 1 shows the chromatic data for the wines analysed. CIELab coordinates a*, b*, and C_ab_* showed significant differences among wines.

a* had negative values for all the samples analysed, revealing a certain greenish tone characterizing PF wines, even though they were relatively small values. b* (and consequently C_ab_*) was the most discriminant variable between the wines analysed. Both a* and b* increased going from non-macerated (W0) to macerated samples, which then appeared more greenish and yellowish [12]. In addition, L decreased when going from W0 to W24 wines, pointing out a corresponding oxidation [13,14]. In the pre-fermentative maceration, the must is in contact with the freshly pressed grape juice, but the alcoholic fermentation has not yet begun. At this stage, the skins are essentially in contact with an aqueous solution, which will mainly determine the extraction of water-soluble substances (e.g., hydroxycinnamic acids and flavans), which, for the presence of high levels of oxygen in the freshly pressed juice, are oxidized, producing *o*-quinones and yellow pigments that give a golden amber tone to the wine. This tendency for the oxidation of macerated wines was also confirmed by the absorbance values recorded at 420 nm (0.08 ± 0.01, 0.12 ± 0.01 and 0.19 ± 0.02, for W0, W18 and W24 samples, respectively; *p* < 0.05), which pointed out a browning of these wines compared to the W0 ones [14,15]. Among macerated wines, prolonged skin contact time (from 18 to 24 h) resulted in a reduction of the CIELab coordinates of a* and b* (−41% and −29%, respectively). This may be due to the secondary oxidative processes that take place with a prolonged maceration. In fact, *o*-quinones (yellow colour and unstable compounds), produced by the enzymatic oxidation of *o*-diphenols, take part in the coupled oxidation reaction with HCAs and flavans generating colourless or slightly yellow pigments [15].

Wine colour differences perceived by the human eye (ΔE_ab*_) [12] were calculated as the Euclidean distance between two points in the three-dimensional space defined by L, a* and b*. For all analysed wines, ΔE_ab*_ values were significantly greater than 2 (*p* < 0.05). The highest value of ΔE_ab*_ was found between W0 and W18 (ΔE_ab*_ = 4.8 ± 0.9), which was higher than three units, indicating that the colour difference between these samples can be easily detected by the observer. Besides, the difference between W18 and W24 samples (2.2 ± 0.9, respectively) was less than three units, making it difficult for the observer to differentiate the colour of these wines.

### 2.2. Bioactive Compound Content and Antiradical Capacity of Passerina del Frusinate Wines

Table 1 shows the flavan and total phenolic content (named FLC and TPC, respectively) of the analysed wines. To the best of our knowledge, there is no literature data on the average TPC and FLC content of PF wines. A comparison between our data on total phenols and that given by the phenol explorer database [16] showed a good similarity for the TPC mean content of PF samples with those reported for rosè wines. Generally, macerated wines were shown to be significantly richer (+54%; *p* < 0.05) in bioactive compounds than in W0 ones. Particularly, they had an average FLC six-fold higher than that of the W0 wines and similar to that normally found for red wines, according to the findings of Fuhrman et al. [17], who showed that processing white wine by imposing a short period of grape skin contact produced polyphenol-rich white wine with antioxidant characteristics similar to those of red wine. On the other hand, Yoo et al. [7] demonstrated that the phenolic enrichment of white wine could impact the taste greatly. In the present study, the catechin concentration of the W18 samples (247.8 mg CAE/L) was generally below the consumer rejection threshold reported by these authors, except in the case of white wines spiked with cathechin-rich green tea extract, for which the rejection threshold of Australian consumers was fixed at a cathechin concentration of 236.4 mg/L. As a consequence, the right balance between healthy properties and taste/flavour perception should be kept in mind for producing health-enhanced white wines acceptable to different cultural consumers.

Data pointed out that longer skin contact times resulted in a reduction of the content of phenolic substances, both in terms of TPC and FLC (−27% and −38%, respectively). This reduction can be attributed to an increased oxidative degradation of the phenolic fraction of W24 wine, which was facilitated by a pH value close to 4 (pH W18: 3.55 ± 0.02; pH W24: 3.99 ± 0.01) as also reported by Olejar et al. [14]. This hypothesis is confirmed by the work of Sant’Anna et al. [18], which showed that the reduction of the brightness of the sample (L) is one of the best indicators from a statistical point of view of the degradation that occurs to the polyphenols and condensed tannins. However, in the present study, only a weak but significant correlation between FLC and L (ρ = −0.439, *p* (two-tailed) < 0.01) was found, while a strong correlation was recorded between FLC and a* (ρ = −0.927, *p* (two-tailed) < 0.01) and an even stronger correlation between FLC and b* (ρ = 0.991, *p* (two-tailed) < 0.01).

The overall antiradical capacity (AC) of wine samples showed the same trend for both synthetic radicals (DPPH^•^ and ABTS^+•^) employed (Table 1). W18 wines were statistically more effective than W24 ones in reducing both the radical targets (*p* < 0.05), and according to its TPC and FLC it was the best scavenger. This can be attributed to the extent of the oxidative degradation of the phenolic fraction of W24 wines, which resulted in a lower AC.

### 2.3. HPLC-DAD Phenolic Acid Fingerprinting

The quantification of total phenolics in wine is usually done by colorimetric methods. Nevertheless, these assays suffer from non-specificity and their main disadvantage is that they only give an estimation of the total phenolic content and they do not give quantitative measurement of individual polyphenol content. For these reasons, in this study, a reversed-phase HPLC-PAD method was developed for the qualitative and quantitative analysis of wine phenolic acids (hydroxybenzoic and hydroxycinnamic acids, namely HBA and HCA, respectively), which are the most important polyphenolic constituents in white wines, both in terms of quantity and ability to participate in redox reactions. The proposed HPLC method was also validated for quantitative purposes (Table 2). Selectivity was checked by using a mixture of available standards, while peak purity was checked by DAD through multivariate analysis using the Agilent Chemstation. Three spectra corresponding to the up slope, apex and down slope of each peak were computer-normalized and superimposed. Peaks were considered pure when the match factor was ≥98%. The intra- (repeatability) and inter-day (reproducibility) precision on compound retention times (t_R_) was evaluated by analysing both a standard mixture and spiked wine samples. Each solution was measured six times in the same day (intra-day precision) and three times a day over four weeks for inter-day precision. Then, the calculated coefficient of variation (CV%) was used to estimate both reproducibility and repeatability. The low variance (0.03–0.29 and 0.18–0.67 for Intra-D and Inter-D, respectively) obtained was good enough for phenolic quantification (Resurreccion, 2009). The LOD (Limit Of Detection) ranged from 0.02 ppm for ferulic acid to 0.88 ppm for gallic acid, while the LOQ (Limit Of Quantification) from 0.05 ppm for ferulic acid to 2.89 ppm for vanillic acid. Ten-point calibration curves based on external standard solutions (0–100 ppm) were generated for each phenolic compound. Linearity was also evaluated by analysing both a standard mixture and spiked wine samples with known amounts of different standard mixture. Pearson’s coefficients obtained by plotting the peak area vs. concentration were ≥0.999, thus confirming the linearity of the method (data not shown).

**Figure 2:**
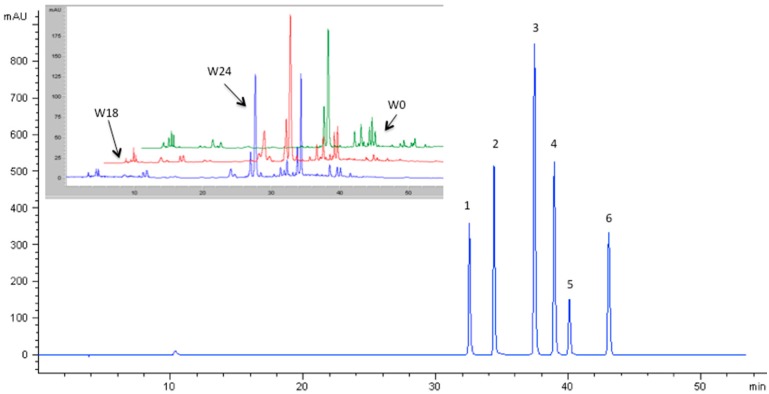
Typical HPLC-DAD chromatograms of phenolic acids and wine samples (in the inset) recorded at 320 nm. 1: caffeic acid; 2: syringic acid; 3: 4-coumaric acid; 4: ferulic acid; 5: 3-coumaric acid; 6: 2-coumaric acid.

Figure 2 shows the typical HPLC-DAD profiles obtained for both phenolic acid standard mixture and wine samples analysed (in the inset of the figure).

As expected, the highest amount of phenolic acids was found in macerated wines, according to the following order: W18 > W24 > W0 (Table 3). W18 samples showed a total phenolic acid content (TFAC) about two times higher than that of the other wines. Regarding the different phenolic acid classes analysed, the total content of HCAs was higher than that of HBAs for all the wines. Maceration led to a higher content of HCAs in the wine regardless of the skin contact time. Moreover, while W18 showed an increase of both HCA and HAB contents compared to that of W0 samples, prolonged skin contact time up to 24 h produced a strong reduction of HBA content (−42%) in W18 samples [12]. The higher content of HCAs of macerated wines agreed with spectral analysis (i.e., CIELab coordinates and 420 nm absorbance values) as previous studies demonstrated that HCAs can be both oxidation substrates and browning precursors, accounting for the higher oxidation potential of wines undergoing a long skin contact time [14]. Results also showed a decrease in almost all phenolic acids analysed with the increasing skin contact time. This can be explained taking into account that oxidative processes play a significant role in changing the phenolic composition of wine since they are accompanied by the oligomerization of the original phenolic compounds.

Table 3 summarizes the content of identified HBAs in the studied samples. Vanillic acid was the most abundant one in the macerated wines, followed by syringic and gallic acids, the latter being the most abundant HBA in W0 samples (Table 3). The identified HBAs represent 5%, 4.4% and 3.5% of the total phenols in W0, W18 and W24 samples, respectively. Among macerated wines, prolonged skin contact time up to 24 h negatively affected the overall HBA content, especially that of vanillic acid, whose concentration was almost halved by prolonged maceration.

Among HCAs, which were higher in macerated samples than in W0 ones, caffeic acid was the most abundant, ranging from 2.36 to 13.4 mg/L (Table 3). This is particularly interesting as free caffeic acid is released by hydrolysis of caftaric acid, a hydroxycinnamate, which during fermentation is oxidized into its principal components. The final concentration of caffeic acid in wine is therefore dependent on oenological practices and this fact can explain the differences observed between macerated and W0 samples. For what concerned the macerated wines, as previously observed for HBA content, prolonged skin contact time negatively affected the overall HCA content of W24 samples, mainly that of caffeic acid (−57.5%), which proved to be the most susceptible to degradation as a result of prolonged maceration. It is a very relevant feature, as Migliori et al. [19] reported that this acid, at the concentrations generally found in white wines, may exert a protective effect on endothelial injury induced by hypoxia and uremic toxins. The reduction of caffeic acid following its oxidation due to prolonged maceration correlates well with the browning of white wines. In fact, correlation analysis pointed out a good correlation between caffeic acid and the CIELab coordinate L (ρ = −0.718, *p* (two-tailed) < 0.01) and between caffeic acid and a* (ρ = −0.860, *p* (two-tailed) < 0.01). Besides, a strong positive correlation was established between caffeic acid and b* (ρ = 0.941, *p* (two-tailed) < 0.01).

## 3. Materials and Methods

### 3.1. Chemicals

For the analysis, four bottles of each wine were analysed and all the reagents used were of analytical spectrophotometric grade (Carlo Erba, Rome, Italy). Folin-Ciocalteu reagent, gallic acid, catechin, 2,2-diphenyl-1-picrylhydrazyl radical (DPPH^•^), vanillin, 2,2’-azinobis-(3-ethylbenzothiazolin-6-sulfonic acid) (ABTS), potassium persulfate, and vanillin were purchased from Sigma-Aldrich (Milan, Italy). Standards used for identification and quantification purposes with HPLC were purchased from Extrasynthese (Genay, France) and Sigma-Aldrich (Milan, Italy). Organic solvents used for chromatography were of HPLC ultragradient grade (Sigma Aldrich, Milan, Italy), while the water employed was previously purified in a Milli-Q system (Millipore, Milan, Italy). The 0.45 μm pore size membrane filters from Pall (Pall Corporation, Ann Arbor, MI, USA) were used for filtration of both mobile phases and samples.

### 3.2. Wine Samples

Mono varietal (100% Passerina cultivar, grown at Piglio (Frosinone, central Italy) (41°49′0″N latitude; 3°07′60″E longitude) commercial white wines (vintages 2013–2014; number of wine samples per year: three wines; number of bottles analysed: four per wine) were obtained by local wineries. All wines were produced using the same winemaking process. Briefly, grapes were destemmed, crashed and then transferred into stainless steel tanks for maceration. The maceration was carried out by control the skin contact conditions (18 and 24 h at 20 °C for W18 and W24 wines, respectively). After maceration, pomaces were softly pressed and sulphur dioxide was added to the obtained must (final concentration: 55 mg/L). No selected yeasts were used. Control wines were produced in the same manner without maceration (W0). At the end of fermentation, the wines were racked and stored in stainless steel tanks to stabilize. Finally, they were filtered and bottled.

### 3.3. Wine Colour Evaluation

Wine colour evaluation was made using a chromameter (CR-400, illuminant D65, 10° standard observer; Minolta, Osaka, Japan) tristimulus colour analyser. All samples were filtrated trough 0.45 m PTFE filters (Millipore, Milan, Italy). The visible reflectance spectra (380–770 nm) were recorded (Δλ = 2 nm) and colour definition was made using the CIELab space, according to Gomez-Miguez et al. [12].

### 3.4. Determination of Total Polyphenol Content

TPC of wine samples was determined using the Folin-Ciocalteu (F-C) method [20], with some modifications as reported by Carbone et al. [21]. TPC was calculated from a calibration curve, using gallic acid as a standard. Results were expressed as milligrams of gallic acid equivalents (GAE) per L of wine (ppm).

### 3.5. Determination of Flavan-3-ols

The vanillin assay was performed as reported by Carbone et al. [21]. FLC was calculated from a calibration curve, using catechin as a standard. Results were expressed as ppm of catechin equivalents (CTE).

### 3.6. Antiradical Capacity Determination

The DPPH^•^ quenching capacity was estimated spectrophotometrically according to Carbone et al. [21]. The decrease in absorbance was measured at 517 nm against a blank (using methanol to replace wine) in a UV-Vis spectrophotometer (Evolution 300, THERMO Scientific, Milan, Italy) at room temperature. EC_50_ was calculated according to Sanchez-Moreno et al. (1998). ABTS^+•^ quenching capacity of wines was estimated according to Carbone and Mencarelli [22] and results were expressed in terms of the percentage decrease of the initial ABTS^•+^ absorption by the wine samples. EC_50_ values were calculated based on dose-response curves.

### 3.7. Chromatographic Analysis of Phenolic Acids Using Diode Array Detection (DAD)

Phenolic acids (PAs) were separated and identified by an analytical high performance liquid chromatography (HPLC) system (Agilent 1100 series, Agilent, Milan, Italy) equipped with a diode array detector (DAD; Agilent Technologies, Milan, Italy). The separation was carried out on a Zorbax SB C18 column (Agilent, 4.6 × 250 mm; 5 μm particle size, set at 30 °C), according to Carbone and Mencarelli (2015). Separated PAs were identified at 280 nm (benzoic acids) and 320 nm (hydroxycinnamic acids), by their retention times, spectral data as compared to individual standards and by the method of standard addition to the samples. Besides, UV-Vis spectra were recorded over the range 200–700 nm. The injection volume was 20 μL and samples were membrane-filtered (Millipore PTFE 0.45 μm, Milan, Italy) before HPLC analysis. Analytical data were evaluated using a software-management system of chromatographic data (Chemstation 32.1, Agilent Technologies). 10-point calibration curves based on external standard solutions (0–100 ppm) were obtained for quantification.

### 3.8. HPLC-DAD Method Validation

The proposed HPLC method for phenolic acid analysis in wines was validated in terms of selectivity, peak purity, precision (intra- and inter-day), linearity, limits of detection (LOD) and quantification (LOQ).

Precision, both intra- and inter-day, was expressed by the coefficient of variation (CV%) of the dataset obtained. The mean peak area and retention time (t_R_) values for each compound were also determined. LOD and LOQ were calculated by the standard deviation (σ) of the y-intercepts of the regression lines and the slope (S), using the following equations:

LOD = 3.3 σ/S(1)

LOQ = 10 σ/S(2)

### 3.9. Statistical Analysis

Statistical analysis was performed with SPSS 17.0 software (SPSS, Inc., Chicago, IL, USA). All measurements were performed at least in triplicate and data were reported as mean ± standard error of the mean (SE). A one-way analysis of variance (ANOVA) was performed on collected data. Bonferroni’s post-hoc test and Pearson’s correlation coefficient (ρ) were used to determine differences between means (*p* < 0.05) and variable correlations (*p* < 0.01).

## 4. Conclusions

For the first time, in the present study we reported some sensorial (e.g., colour characteristics) and nutraceutical features of 100% Passerina del Frusinate white wine. Results highlight the good antiradical and poliphenolic potential of this wine, which is similar to that found in rosè wines. The present findings also indicate that the use of prolonged skin contact has a great impact on both the colour and the overall nutraceutical properties of wines. Extended maceration up to 24 h produces wine browning, with a strong reduction of the CIELab coordinates. Moreover, the maceration length also appears as a critical parameter for the nutraceutical and antiradical potential of wines, having a great impact on phenolic acids, mainly vanillic and caffeic ones. Prolonged skin contact up to 24 h strongly reduces the content of these acids, the concentrations of which are halved.

Finally, the research findings indicate that the vinification process may benefit from maceration with extended skin contact up to 18 h, which has the potential to increase the content of the compounds that are important for wine production and quality, such as phenolic acids.

## Figures and Tables

**Table 1:** Phytochemical content and antiradical capacity of wines analysed (mean ± SE).

Sample	TPC ^1^	FLC ^2^	AC_DPPH_^3^	AC_ABTS_^4^
W0	391 ± 2 ^a^	32.7 ± 0.3 ^a^	37 ± 2 ^b^	34 ± 0.6 ^b^
W18	695 ± 5 ^c^	247.8 ± 0.8 ^c^	25.9 ± 0.4 ^a^	28.0 ± 0.9 ^a^
W24	509 ± 4 ^b^	154.0 ± 0.5 ^b^	36 ± 1 ^b^	36 ± 1 ^b^

Experimental number: 24 samples. ^1^ TPC: total polyphenol content expressed as mg GAE/L; ^2^ FLC: total flavan-3-ol content expressed as mg CAE/L; ^3^ AC_DPPH_: antiradical capacity expressed in terms of EC_50_ = μL of wine required to obtain 50% DPPH• scavenging; ^4^ AC_ABTS_: antiradical capacity expressed in terms of EC_50_ = μL of wine required to obtain 50% ABTS^+^. Significant differences between groups are shown with different letters according to Bonferroni’s post-hoc test (*p* < 0.05).

**Table 2:** Validation parameters for the analysed bioactive compounds using pure standards.

Chemical Class	Compound	Retention Time (min)	Intra-D (CV%)	Inter-D (CV%)	LOD ^1^ (ppm)	LOQ ^2^ (ppm)
HBAs ^3^	Gallic acid	10.31 ± 0.03	0.29	0.67	0.88	2.66
	Vanillic acid	33.35 ± 0.02	0.06	0.33	0.95	2.89
	Syringic acid	34.42 ± 0.01	0.03	0.18	0.10	0.29
HCAs ^4^	Caffeic acid	33.71 ± 0.01	0.04	0.20	0.36	1.11
	4-coumaric acid	37.66 ± 0.01	0.04	0.18	0.57	1.34
	Ferulic acid	39.06 ± 0.01	0.03	0.18	0.02	0.05
	3-coumaric acid	40.16 ± 0.02	0.05	0.21	0.25	0.77
	2-coumaric acid	43.08 ± 0.02	0.05	0.28	0.36	1.09

^1^ LOD: limit of detection; ^2^ LOQ: limit of quantitation; ^3^ HBAs: hydroxybenzoic acids; ^4^ HCAs: hydroxycinnamic acids.

**Table 3:** Concentration (mg/L) of phenolic compounds determined by HPLC-DAD in the analysed wines (mean ± SE).

Chemical Class	Compound	W0	W18	W24
HBAs ^1^	gallic acid	10.6 ± 0.1 ^c^	5.9 ± 0.1 ^b^	3.42 ± 0.04 ^a^
	vanillic acid	5.23 ± 0.05 ^a^	16.2 ± 0.2 ^c^	8.5 ± 0.2 ^b^
	syringic acid	3.30 ± 0.01 ^a^	8.5 ± 0.2 ^c^	5.9 ± 0.1 ^b^
HCAs ^2^	caffeic acid	2.36 ± 0.02 ^a^	13.4 ± 0.1 ^c^	5.69 ± 0.04 ^b^
	4-coumaric acid	1.75 ± 0.01 ^b^	1.41 ± 0.04 ^a^	1.94 ± 0.01 ^c^
	ferulic acid	1.40 ± 0.01 ^a^	1.39 ± 0.02 ^a^	1.75 ± 0.02 ^b^
	3-coumaric acid	trace	trace	nd
	2-coumaric acid	trace	trace	nd

Experimental number: 24 samples. ^1^ HBAs: hydroxybenzoic acids; ^2^ HCAs: hydroxycinnamic acids. nd: not detectable. Trace is below the LOQ but above the LOD. In a row, significant differences are shown with different letters according to Bonferroni’s post-hoc test (*p* < 0.05).
